# Prevalence of Misinformation and Factchecks on the COVID-19 Pandemic in 35 Countries: Observational Infodemiology Study

**DOI:** 10.2196/23279

**Published:** 2021-02-13

**Authors:** Meeyoung Cha, Chiyoung Cha, Karandeep Singh, Gabriel Lima, Yong-Yeol Ahn, Juhi Kulshrestha, Onur Varol

**Affiliations:** 1 School of Computing Korea Advanced Institute of Science and Technology Daejeon Republic of Korea; 2 Data Science Group Institute for Basic Science Daejeon Republic of Korea; 3 College of Nursing Ewha Womans University Seoul Republic of Korea; 4 Center for Complex Networks and Systems Research Luddy School of Informatics, Computing, and Engineering Indiana University Bloomington, IN United States; 5 Indiana University Network Science Institute Indiana University Bloomington, IN United States; 6 Connection Science Massachusetts Institute of Technology Cambridge, MA United States; 7 GESIS - Leibniz Institute for the Social Sciences Cologne Germany; 8 Computer Science Department Sabanci University Istanbul Turkey

**Keywords:** COVID-19, coronavirus, infodemic, infodemiology, misinformation, vulnerability, LMIC countries

## Abstract

**Background:**

The COVID-19 pandemic has been accompanied by an infodemic, in which a plethora of false information has been rapidly disseminated online, leading to serious harm worldwide.

**Objective:**

This study aims to analyze the prevalence of common misinformation related to the COVID-19 pandemic.

**Methods:**

We conducted an online survey via social media platforms and a survey company to determine whether respondents have been exposed to a broad set of false claims and fact-checked information on the disease.

**Results:**

We obtained more than 41,000 responses from 1257 participants in 85 countries, but for our analysis, we only included responses from 35 countries that had at least 15 respondents. We identified a strong negative correlation between a country’s Gross Domestic Product per-capita and the prevalence of misinformation, with poorer countries having a higher prevalence of misinformation (Spearman ρ=–0.72; *P*<.001). We also found that fact checks spread to a lesser degree than their respective false claims, following a sublinear trend (β=.64).

**Conclusions:**

Our results imply that the potential harm of misinformation could be more substantial for low-income countries than high-income countries. Countries with poor infrastructures might have to combat not only the spreading pandemic but also the COVID-19 infodemic, which can derail efforts in saving lives.

## Introduction

COVID-19, caused by SARS-CoV-2 [1], has spread worldwide, becoming a global pandemic. Most preventive measures against the disease comprise individual behaviors, as therapeutics are under development and yet to be approved by national health agencies [2]. Since such measures require individuals to change their behaviors according to validated information about the disease, effective communication of accurate information to the public is critical for minimizing the pandemic’s impact. However, as observed previously in the context of the anti-vaccination movement [3], communicating accurate health-related information can be challenging. Moreover, social media can rapidly disseminate a piece of misinformation about the disease to millions of people. The amplifying nature of online platforms can threaten public health by creating an *infodemic* [4].

The COVID-19 infodemic has shown to be exceptionally harmful in the context of both individual and public health. For instance, misinformation has motivated people to attack and abuse health care workers [5]. There have been reports of eggs being thrown at nurses in Mexico [6] and Indian doctors being evicted from their houses under the belief that they were vectors of the disease [7]. An even worse event has occurred in Iran, where over a hundred people died and thousands became severely ill due to methanol poisoning [8]. The infodemic has also had negative consequences on the psychosocial health of various layers of society. Widespread misinformation has affected the global population by increasing levels of depression, anxiety, and posttraumatic stress disorder [9].

The negative health impact of the infodemic could also widen the already extensive world health literacy gap. People with low health literacy are more vulnerable to an infodemic, as they tend to have a limited ability to seek, comprehend, and evaluate health information from social media [10]. Research has found a strong relationship between health literacy and adverse health outcomes from infectious diseases [11]. Therefore, assessing the level of eHealth literacy on COVID-19 and understanding how the COVID-19 infodemic has spread worldwide are crucial efforts in the current pandemic [12].

Many of the infodemic’s claims have been locally fact-checked. However, debunking information does not spread effectively and rapidly enough through the global population, leading to misinformation and causing further harm in other parts of the world [13]. Prior research has shown that false news spreads faster than its factual counterparts [14]. Hence, misinformation could gain a strong foothold over their trustworthy counterparts, and the current COVID-19 infodemic might prove to be especially harmful, as it tackles health-related behaviors that could lead to life and death consequences.

To prevent the harm caused by misinformation, we have launched “Facts Before Rumors.” This has been a pre-emptive public communication campaign to combat COVID-19 misinformation by spreading fact-checked information from countries that have seen false information earlier to regions that have not necessarily seen the same piece of information yet. Our campaign’s distinguishing feature is that the current project is proactively propagating validated responses to claims seen in other countries and regions, thereby pre-emptively suppressing the dissemination of false health information. Alongside our campaign, we have also conducted a survey-based study to quantify the infodemic’s reach worldwide. Specifically, we investigated the public exposure to false claims and fact-checked information across different world regions. We present our findings in the following sections.

## Methods

After identifying more than 200 claims about COVID-19 that had been fact-checked in China, duplicated claims, rumors not related to health (eg, political conspiracies), and claims addressing local topics were removed. Two Chinese-speaking researchers were involved in this process. In total, 11 pieces of misinformation addressing health-related behaviors were chosen:

1. Hot: The virus will only spread in cold, dry weather and does not survive in hot, humid weather.
2. Sauna: Hot baths or saunas can reduce the chances of getting infected with COVID-19.
3. Drink: Drinking water or tea frequently will cure a COVID-19 infection.
4. Mask: Microwave, steam, blow-dry, or spray alcohol to clean used face masks.
5. Garlic: Garlic, ginger, onion, sesame oil, probiotics, herbal remedies, or aromatherapy can prevent the infection.
6. Dryer: Hot air dryers can kill the virus.
7. Salt: Gargling with salt water, vinegar, or saline nose rinse can eliminate the virus.
8. Age: Only certain age groups, races, and ethnicities are vulnerable to the virus.
9. Test: You can test yourself for COVID-19 by holding your breath for 10 seconds.
10. Eggs: Eating eggs every day can cure the virus.
11. Bleach: Spraying alcohol or chlorine over your body will kill the virus.

We conducted a large-scale online survey via Pollfish, a survey company, and personal social media channels. The respondents were recruited via convenience sampling, given the large-scale nature of the study. Pollfish conducts surveys by randomizing its delivery to the targeted populations via mobile apps. Respondents were compensated with nonmonetary incentives such as extra lives in a game or access to premium content. As per the documentation, Pollfish has partnerships with over 120,000 app providers and is present in over 160 countries worldwide. We obtained more than 41,000 responses from 1257 unique individuals residing in 85 countries between early April and mid-May 2020.

In our study, we asked whether participants have seen the chosen claims, whether they believed that exposing fact-checked information of those claims would be beneficial to their community, and whether these claims have been either confirmed or denied by official sources. The respondents also reported their perceived financial and health status, alongside various demographic questions, such as sex and age. For analysis, we only included 35 countries that had at least 15 respondents to eliminate noisy and biased observations for those countries.

Participant recruitment relied on the survey platform’s methodology, and the only demographic control added was age (ie, older than 18 years). To the survey question “How would you rate your financial status?” participants on average reported a similar level of perceived financial status near the response category “fair” among “very poor,” “poor,” “fair,” “good,” and “excellent,” (mean 0.268, median 0, when converted to a 5-point bipolar scale). Hence, we consider that respondents from distinct countries belong to similar economic classes. We report the demographic distribution of survey participants in Table 1.

**Table 1:** Demographic attributes of survey participants (N=1257).

Demographic attributes		Participants, n (%)
**Gender**		
	Female	499 (39.70)
	Male	750 (59.67)
	Other	8 (0.63)
**Age groups (years)**		
	18-24	399 (31.74)
	25-34	422 (33.57)
	35-44	282 (22.43)
	44-54	97 (7.71)
	55-64	48 (3.81)
	≥65	9 (0.70)
**Education**		
	High school	521 (41.45)
	University or college	409 (32.53)
	Graduate school or more	327 (26.01)
**Health status**		
	Very poor	14 (1.11)
	Poor	42 (3.34)
	Fair	205 (16.30)
	Good	616 (49.01)
	Very good	380 (30.23)
**Financial status**		
	Very poor	47 (3.74)
	Poor	169 (13.44)
	Fair	531 (42.24)
	Good	420 (33.41)
	Excellent	90 (7.16)

## Results

Although some false claims were geographically confined, our survey revealed that many false claims recurred across different countries and languages, highlighting the far-reaching power of the infodemic.

For instance, the claim stating that eating eggs every day could cure the disease has primarily spread across Asia. In contrast, the claim stating that SARS-CoV-2 would only spread under cold and dry weather was seen (in its entirety or partly) by more than 82% of our total respondents across all continents.

**Figure 1:**
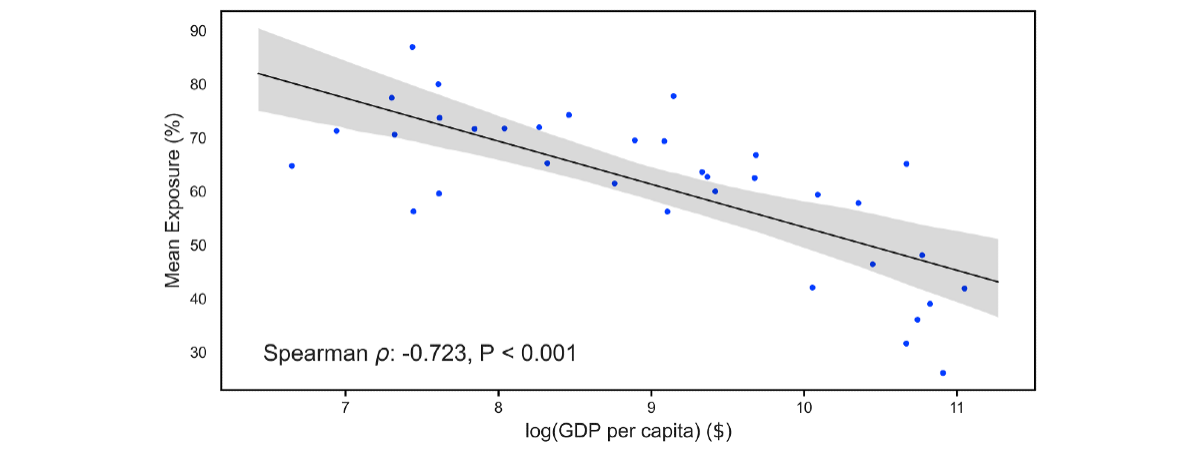
Exposure rate of the selected 11 claims across different countries. Results are shown for countries with at least 15 survey participants. The x-axis indicates the log of GDP per capita of different countries. The countries in the increasing order of GDP per capita are Ethiopia, Nepal, Pakistan, Cambodia, Bangladesh, Kenya, India, Nicaragua, Nigeria, Egypt, Philippines, Indonesia, Sri Lanka, Azerbaijan, South Africa, Thailand, Cuba, Brazil, Turkey, Russia, Argentina, Romania, Chile, Venezuela, Estonia, Bahrain, South Korea, Italy, the United Kingdom, the United Arab Emirates, Canada, Germany, Finland, Sweden, and the United States. The y-axis indicates the average percentage of claims that respondents had seen (ie, mean exposure to claims in a country). GDP: gross domestic product.

When comparing the exposure to false claims across countries, we found that countries with lower gross domestic product per capita, which are likely much more vulnerable to the disease itself, tend to exhibit higher rates of exposure to false claims (see Figure 1; Spearman *ρ*=–0.72; *P*<.001). Our observation indicates that these false claims could particularly hit countries or groups of people with limited access to information even harder, compounding the finding that poorer countries are more vulnerable to communicable diseases [15].

**Figure 2:**
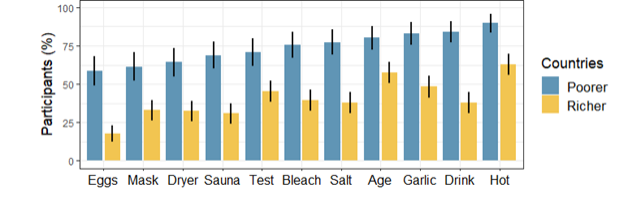
Exposure rate of the selected 11 claims in the poorest and wealthiest countries. Rates are shown for the bottom (Ethiopia, Nepal, Pakistan, Cambodia, Bangladesh) and top five (the United States, Sweden, Finland, Germany, Canada) countries in terms of gross domestic product per capita. The bars depict the mean percentage of respondents who have at least partially seen each claim and standard error bars. Claims on the x-axis are sorted by mean claim exposure rate in the poorest countries.

Figure 2 depicts the average exposure rates for the eleven claims for the top and bottom five countries ranked by gross domestic product per capita. It exemplifies how different rumors have distinct spread patterns worldwide. Unlike the actual disease, which first invaded richer countries, the infodemic that follows it is attacking, via the well-connected internet communities, vulnerable countries the most.

Our results also indicated that those countries with a higher incidence of false claims have also had a substantial amount of fact checks debunking this information. However, this relationship is sublinear; for an increase of 1% in citizens seeing our selected claims, marginally more than half (*β*=.64) of them would have also been presented with debunking information. Therefore, countries most affected by the COVID-19 misinformation rapidly encounter false information that is not fact-checked by official sources at the same rate. This is a concern as people are less active in seeking personal health strategies [16].

Furthermore, people who had been previously exposed to COVID-19 claims perceived a more significant benefit in sharing fact-checked information of claims (Pearson *r*=0.44; *P*<.001). This means that campaigns such as ours would be viewed as valuable, particularly in countries currently experiencing misinformation at a higher degree.

## Discussion

Our results highlight that low-income countries may be at a higher risk of exposure to misinformation and be disadvantaged by the COVID-19 infodemic during the global pandemic. Fact-checked information does not propagate at the same speed as false information, and therefore, countries most affected by the infodemic should also have a higher incidence of unchecked information. Our results warrant a pre-emptive strategy for busting misinformation and indicate a higher demand for localized fact checks in these countries and a public belief, especially in low-income countries, that fact-checking campaigns can benefit their local community.

The analysis presented in this study has some limitations. Although we conducted a large-scale survey to quantify the spread of misinformation in different countries, our sample was not necessarily representative of the target countries’ population. Additionally, our survey covered respondents from 35 countries, and our analysis did not consider other parts of the world. Moreover, we have included 11 health-related false claims that circulated online in China during the pandemic’s infancy. Future work could address a broader range of rumors, such as from political topics, as we have chosen to not tackle them in this study. We have also not obtained information about how respondents were exposed to claims, such as through social media platforms or traditional media.

In spite of the aforementioned limitations, the current analysis results have yielded interesting and useful insights into the spread of misinformation across different countries of the world. As future work, we plan to propagate our campaign to a broader audience in more countries to suppress the infodemic proactively and extend our results from this and upcoming studies to more representative samples and other demographic variables.
